# Inhibition of OCT4 binding at the *MYCN* locus induces neuroblastoma cell death accompanied by downregulation of transcripts with high-open reading frame dominance

**DOI:** 10.3389/fonc.2024.1237378

**Published:** 2024-02-08

**Authors:** Kazuma Nakatani, Hiroyuki Kogashi, Takanori Miyamoto, Taiki Setoguchi, Tetsushi Sakuma, Kazuto Kugou, Yoshinori Hasegawa, Takashi Yamamoto, Yoshitaka Hippo, Yusuke Suenaga

**Affiliations:** ^1^ Laboratory of Evolutionary Oncology, Chiba Cancer Center Research Institute, Chiba, Japan; ^2^ Graduate School of Medical and Pharmaceutical Sciences, Chiba University, Chiba, Japan; ^3^ Innovative Medicine CHIBA Doctoral WISE Program, Chiba University, Chiba, Japan; ^4^ All Directional Innovation Creator Ph.D. Project, Chiba University, Chiba, Japan; ^5^ Department of Neurosurgery, Chiba Cancer Center, Chiba, Japan; ^6^ Graduate School of Integrated Sciences for Life, Hiroshima University, Hiroshima, Japan; ^7^ Department of Applied Genomics, Kazusa DNA Research Institute, Chiba, Japan; ^8^ Laboratory of Precision Tumor Model Systems, Chiba Cancer Center Research Institute, Chiba, Japan

**Keywords:** neuroblastoma, MYCN, oct4, open reading frame dominance, CRISPR/dCas9, p53, MDM2, caspase-2

## Abstract

Amplification of *MYCN* is observed in high-risk neuroblastomas (NBs) and is associated with a poor prognosis. *MYCN* expression is directly regulated by multiple transcription factors, including OCT4, MYCN, CTCF, and p53 in NB. Our previous study showed that inhibition of p53 binding at the *MYCN* locus induces NB cell death. However, it remains unclear whether inhibition of alternative transcription factor induces NB cell death. In this study, we revealed that the inhibition of OCT4 binding at the *MYCN* locus, a critical site for the human-specific OCT4–MYCN positive feedback loop, induces caspase-2-mediated cell death in *MYCN*-amplified NB. We used the CRISPR/deactivated Cas9 (dCas9) technology to specifically inhibit transcription factors from binding to the *MYCN* locus in the *MYCN*-amplified NB cell lines CHP134 and IMR32. In both cell lines, the inhibition of OCT4 binding at the *MYCN* locus reduced MYCN expression, thereby suppressing MYCN-target genes. After inhibition of OCT4 binding, differentially downregulated transcripts were associated with high-open reading frame (ORF) dominance score, which is associated with the translation efficiency of transcripts. These transcripts were enriched in splicing factors, including MYCN-target genes such as *HNRNPA1* and *PTBP1*. Furthermore, transcripts with a high-ORF dominance score were significantly associated with genes whose high expression is associated with a poor prognosis in NB. Because the ORF dominance score correlates with the translation efficiency of transcripts, our findings suggest that MYCN maintains the expression of transcripts with high translation efficiency, contributing to a poor prognosis in NB. In conclusion, the inhibition of OCT4 binding at the *MYCN* locus resulted in reduced MYCN activity, which in turn led to the downregulation of high-ORF dominance transcripts and subsequently induced caspase-2-mediated cell death in *MYCN*-amplified NB cells. Therefore, disruption of the OCT4 binding at the *MYCN* locus may serve as an effective therapeutic strategy for *MYCN*-amplified NB.

## 1 Introduction

Neuroblastoma (NB) is the most common extracranial solid tumor in children, accounting for 12%–15% of all cancer-related deaths in children (1–3). At least 40% of all NBs are designated as high-risk tumors and often show *MYCN* amplification (4). Amplification of *MYCN* is observed in 25% of high-risk cases and correlates with poor clinical outcomes in patients with NB (5, 6). *Th-MYCN* mice, which are used as a preclinical *in vivo* model of NB, spontaneously develop NB, highlighting the significance of *MYCN* as a potent oncogene in the pathogenesis of NB (7). Despite current therapeutic advances, therapeutic strategy for targeting MYCN remains a medical challenge (8). Therefore, new MYCN-targeting therapeutic strategies are required to further improve patient outcomes.

MYCN, a basic helix–loop–helix transcription factor, directly regulates the transcription of genes involved in diverse cellular processes, such as cell growth, apoptosis, and differentiation (4). It directly binds to its own intron 1 region and upregulates its own expression and its *cis*-antisense gene *NCYM* by forming a positive autoregulatory loop in NB cells (9–11). In addition to MYCN, other transcription factors bind to the *MYCN* locus to regulate *MYCN* expression in NB. For example, OCT4, a transcription factor that maintains cancer stemness, is highly expressed in NB, regulates multipotency, and contributes to drug-resistant phenotypes of NB (12–17). In our previous study, we found that OCT4 stimulates *MYCN* transcription by binding to the intron 1 of *MYCN* locus, whereas MYCN stimulates *OCT4* transcription by binding to the *OCT4* promoter region (17). The OCT4-binding sequence in intron 1 of *MYCN* is not present in mice but mostly conserved in other mammals (17). In contrast, the E-box in the MYCN-binding region of the *OCT4* promoter is specific to humans and absent even in chimpanzees (17). Thus, OCT4 and MYCN form a human-specific positive feedback loop in NB (17). This human-specific positive feedback loop contributes to the stemness of *MYCN*-amplified NB by maintaining the expression of stem cell-related genes including *LIN28*, *NANOG*, and *SOX2* (17). Additionally, CCCTC-binding factor (CTCF), an insulator protein that is capable of regulating gene expression, stimulates *MYCN* transcription by binding to the *MYCN* promoter region (18). Previous studies investigated the transcriptional regulation of *MYCN* through knockout/knockdown of upstream transcription factors. However, since the expression level of transcription factors themselves are reduced by this method, the expression of downstream genes other than *MYCN* is also altered, and indirect effects of such changes on *MYCN* expression cannot be ruled out. In addition, overexpression of upstream factors used in previous studies is based on expression levels of transcription factors that are not observed under normal physiological conditions. In particular, it is necessary to reevaluate whether binding of CTCF to the *MYCN* region is essential for the *MYCN* expression because CTCF functions as an insulator and affects chromatin status of the entire genome. Therefore, the significance of binding of these transcription factors on *MYCN* locus for regulation of *MYCN* transcription has remained elusive. Recently, a CRISPR/deactivated cas9 (dCas9) system has been developed to specifically inhibit binding of transcription factors without affecting their intrinsic expression levels (19). We previously reported that blocking the p53-binding site on *MYCN* locus using the CRISPR/dCas9 system upregulates *MYCN*, *NCYM*, and p53 expression, inducing apoptotic cell death accompanied by caspase-2 activation (20). Thus, the p53-mediated repression of MYCN/NCYM contributes to the survival of *MYCN*-amplified NB cells (11, 20). However, it remains unclear whether the binding of other transcription factors (OCT4, MYCN, and CTCF) at the *MYCN* locus affects *MYCN* expression and contributes to NB cell survival.

In this study, we evaluated the significance of transcription factors that bind to the *MYCN* locus in NB cells. Our results suggest that the OCT4 binding at the *MYCN* locus plays a crucial role in *MYCN*-amplified NB cell survival.

## 2 Material and methods

### 2.1 Cell culture

Human NB cell lines CHP134 and IMR32 were maintained in RPMI-1640 (Nacalai Tesque, Kyoto, Japan) supplemented with 10% fetal bovine serum (Thermo Fisher Scientific, Waltham, MA), 50 U/mL penicillin, and 50 μg/mL streptomycin (Thermo Fisher Scientific, Waltham, MA). Neuroblastoma cell line SK-N-AS was maintained in Dulbecco’s Modified Eagle Medium (Sigma-Aldrich, St. Louis, MO) supplemented with 10% fetal bovine serum (Thermo Fisher Scientific, Waltham, MA), 50 U/mL penicillin, and 50 μg/mL streptomycin (Thermo Fisher Scientific, Waltham, MA).

### 2.2 Vector construction

To inhibit transcription factor binding at the *MYCN* locus, we designed CRISPR guide RNAs against the MYCN-binding site (9, 10), OCT4-binding site (17), CTCF-binding site A (18), p53-binding site (20), and CTCF-binding site B (data from the UCSC Genome Browser). A CRISPR/dCas9 vector was constructed as follows: pX330A_dCas9-1x2 (Addgene, Watertown, MA; plasmid ID 63596) (21) was treated with BpiI (Thermo Fisher Scientific, Waltham, MA). Thereafter, annealed oligonucleotides (p53-binding site: sense: 5′-CACCGCGCCTGGCTAGCGCTTGCT-3′, antisense: 5′-AAACAGCAAG CGCTAGCCAGGCGC-3′; OCT4-binding site: sense: 5′-CACC AGCAGGGCTTGCAAACCGCC-3′, antisense: 5′-AAACGGCGGTTTGCAAGCCCTGCT-3′; MYCN-binding site: sense: 5′-CACC GGGAGGGGGCATGCAGATGC-3′, antisense: 5′-AAAC GCATCTGCATGCCCCCTCCC-3′; CTCF-A-binding site: sense: 5′-CACC TCTCCGCGAGGTGTCGCCTT-3′, antisense: 5′-AAACAAGGCGACACCTCGCGGAGA-3′; and CTCF-B-binding site: sense: 5′-CACCCCAGCAGGCGGCGATATGCG-3′, antisense: 5′-AAACCGCATATCGCCGCCTGCTGG-3′) were inserted into the digested vector.

### 2.3 Transfection

Plasmid transfection was performed using the Neon Transfection System (Invitrogen, Carlsbad, CA) according to the manufacturer’s instructions. We used 2 × 10^5^ cells and 4 μg of the plasmid per transfection. When performing the CUT&RUN assay and RNA isolation for quantitative real-time reverse transcription-polymerase chain reaction (qRT-PCR), plasmid transfections were performed using Lipofectamine 3000 transfection reagent (Invitrogen, Carlsbad, CA), according to the manufacturer’s instructions.

### 2.4 WST assay

Cell proliferation was evaluated using the Cell Counting Kit-8 (CCK-8; Dojindo Laboratories, Kumamoto, Japan), according to the manufacturer’s protocol. Briefly, 100 μL of dCas9-transfected cell suspension (5,000 cells/well) was seeded in a 96-well plate. Ninety-six hours after transfection of CRISPR/dCas9, 10 μL of CCK-8 reagent was added into each well of the 96-well plate, and then, the cells were incubated for 2 h at 37°C in a 5% CO_2_ incubator. Cell proliferation was monitored at 450 nm using CORONA absorbance microplate reader (MTP-310, CORONA ELECTRIC, Ibaraki, Japan).

### 2.5 Cytotoxicity assay

To evaluate cell damage, we measured lactate dehydrogenase (LDH) activity released from cells. LDH activity was measured using the LDH Cytotoxicity Assay Kit (Nacalai Tesque, Kyoto, Japan), according to the manufacturer’s instructions. Briefly, 100 μL of dCas9-transfected cell suspension (10,000 cells/well) was seeded in a 96-well plate. Ninety-six hours after transfection of CRISPR/dCas9, 100 μL of the substrate solution was added into each well of the 96-well plate. After which, the cells were incubated for 20 min at room temperature under shading condition, and then, 50 μL of the stop solution was added into each well of the 96-well plate. LDH activity was monitored at 490 nm using 2030 ARVO X (PerkinElmer, Kanagawa, Japan).

### 2.6 CUT&RUN assay

Twenty-four hours after the transfection of CRISPR/dCas9, CUT&RUN (CUT&RUN Assay Kit, #86652, Cell Signaling Technology, Danvers, MA) was performed according to the manufacturer’s instructions. For each reaction, 1 × 10^5^ cells were used, and the cells were bound to concanavalin A beads and permeabilized with a digitonin-containing buffer. Antibodies were then added and incubated at 4°C for 2 h. The following antibodies were used in the assay: anti-OCT4 antibody (15 μL/assay, #2750; Cell Signaling Technology, Danvers, MA), anti-RNA Pol II antibody (5 μL/assay, #14958; Cell Signaling Technology, Danvers, MA), anti-RNA PolII C-terminal domain (CTD) phospho Ser2 (Pol II pSer2) antibody (5 μL/assay, # 13499; Cell Signaling Technology, Danvers, MA), anti-RNA PolIICTD phospho Ser5 (Pol II pSer5) antibody (5 μL/assay, # 13523; Cell Signaling Technology, Danvers, MA), and Rabbit (DA1E) mAb IgG XP^®^ Isotype Control (#66362; Cell Signaling Technology, Danvers, MA). As an isotype control for the anti-OCT4 antibody, 15 μL of the anti-IgG antibody was applied per assay. In the case of other antibodies, 5 μL of the anti-IgG antibody was applied per assay. DNA obtained from the CUT&RUN assay was amplified using SYBR Green qRT-PCR with the StepOnePlus™ Real-Time PCR System (Thermo Fisher Scientific, Waltham, MA). The following primer set was used: Primer #1, forward 5′-TCCTGGGAACTGTGTTGGAG-3′ and reverse 5′-CTCGGATGGCTACAGTCTGT -3′; Primer #2, forward 5′-CCCTAATCCTTTTGCAGCCC-3′and reverse 5′-CCGACAGCTCAAACACAGAC-3′; Primer #1 in **Figure S4C**, forward 5′-TCCTGGGAACTGTGTTGGAG-3′ and reverse 5′-CTCGGATGGCTACAGTCTGT-3′; Primer #2 in **Figure S4C**, forward 5′-ACTGTAGCCATCCGAGGACA-3′ and reverse 5′-CAAGCCCTGCTCCTTACCTC-3′; Primer #3 in **Figure S4C**, forward 5′-CTAATATGCCCGGGGGACTG-3′ and reverse 5′-CTCTAGCCAGGATGCCTTCG-3′; Primer #4 in **Figure S4C**, forward 5′-CCCTAATCCTTTTGCAGCCC-3′ and reverse 5′-CCGACAGCTCAAACACAGAC-3′; Primer #5 in **Figure S4C**, forward 5′-CGTGCTCGTGAGAGCTAGAA-3′ and reverse 5′-GGCTCCGCAACTTTGGAAAC-3′; Primer #6 in **Figure S4C**, forward 5′-GTGTCTGTCGGTTGCAGTGT-3′ and reverse 5′-TTAATACCGGGGGTGCTTCC-3′; and Primer #7 in **Figure S4C**, forward 5′-GGGCATGATCTGCAAGAACC-3′ and reverse 5′-GAAGTCATCTTCGTCCGGGT-3′.The detected DNA levels were normalized by the input signal.

### 2.7 RNA isolation and qRT-PCR

One day after CRISPR/dCas9 transfection, the total RNA from dCas9-transfected NB cells was isolated using the RNeasy Mini Kit (Qiagen, Hilden, Germany) following the manufacturer’s instructions. cDNA was synthesized using SuperScript II with random primers (Invitrogen, Carlsbad, CA). qRT-PCR was performed using SYBR Green PCR with the StepOnePlus™ Real-Time PCR System (Thermo Fisher Scientific, Waltham, MA). The following primer sets were used: *MYCN*, Primer #3, forward: 5′-TCCATGACAGCGCTAAACGTT-3′ and reverse: 5′-GGAACACACAAGGTGACTTCAACA-3′ and *OCT4*, forward: 5′- GGGTTTTTGGGATTAAGTTCTTC-3′, and reverse: 5′- GCCCCCACCCTTTGTGTT-3′ and *GAPDH*, forward: 5′-GTCTCCTCTGACTTCAACAGCG-3′ and reverse: 5′-ACCACCCTGTTGCTGTAGCCAA-3′. *β-Actin* expression was quantified using the TaqMan real-time PCR assay. The mRNA level of *MYCN* was normalized by *β-Actin* and *GAPDH*.

### 2.8 Long-read and short-read RNA sequencing

Twenty-four hours after CRISPR/dCas9 transfection, the total RNA from dCas9-transfected NB cells was isolated using the RNeasy Mini Kit (Qiagen, Hilden, Germany) following the manufacturer’s instructions. An Iso-Seq library was prepared as described in the Procedure & Checklist-Iso-Seq Express Template Preparation for Sequel and Sequel II Systems, Version 02, October 2019 (Pacific Biosciences, Menlo Park, CA). Briefly, cDNA was synthesized and amplified using the NEBNext Single Cell/Low Input cDNA Synthesis & Amplification Module (New England Biolabs, Ipswich, MA), Iso-Seq Express Oligo Kit (Pacific Biosciences, Menlo Park, CA), and barcoded primers. The size of the amplified cDNA was selected using ProNex beads (Promega, Madison, WI) under standard conditions. The Iso-Seq library was prepared from the size-selected cDNA using SMRTbell Express Template Prep Kit 2.0 (Pacific Biosciences, Menlo Park, CA). The Iso-Seq libraries were sequenced on the PacBio Sequel IIe with Sequel ICS v11.0 for 24 h using a single cell of Sequel II SMRT Cell 8M Tray, Sequel II Sequencing Kit 2.0, Sequel II Binding Kit 2.1, and Internal Control 1.0 (Pacific Biosciences, Menlo Park, CA). Circular consensus sequencing (CCS) reads were created using this instrument. An RNA-sequencing (RNA-seq) library was prepared using the NEBNext rRNA Depletion Kit v2 (Human/Mouse/Rat) and the NEBNext Ultra II Directional RNA Library Prep Kit for Illumina (New England Biolabs, Ipswich, MA). The RNA-seq libraries were sequenced on NextSeq 500 using the NextSeq 500/550 High Output Kit v2.5 (75 cycles) (Illumina, San Diego, CA).

### 2.9 Bioinformatic analysis

Demultiplexing of CCS reads and removal of cDNA primers were performed using the lima command of SMRT Tools v11.0 (Pacific Biosciences, Menlo Park, CA) with the parameters of Iso-Seq data. Removing of artificial concatemer and reads without polyA tail or with short polyA (less than 20 nt) and trimming of polyA tail were performed using the isoseq3 refine tool with the “–require-polya” parameter. High-quality isoforms were obtained using the isoseq3 cluster with the ‘–use-qvs’ parameter. To collapse the transcripts using the isoseq3 collapse command, the high-quality isoform reads were aligned to the human genome GRCh38 using minimap2 v2.24 (22) with the parameter –preset ISOSEQ. Quality control and filtering of the collapsed transcripts were performed using SQANTI3 (23) with genome annotation (Ensembl GRCh38 release105) to remove 3’-end intrapriming artifact, RT-switching artifact, and low frequency transcript (less than 2 fragments). To identify novel transcripts and remove transcript redundancy in all samples, the filtered transcripts were compared with known transcripts (Ensembl GRCh38 release105) using the TALON v5.0 pipeline (24) with the “–cov 0.95 –identity 0.95 –observed” parameter. Transcript reference sequences, including novel and known transcript sequences, were created using SQANTI3 and used in the following short-read RNA-seq analysis. Salmon v1.9.0 was used to quantify transcript expression levels with the “fldMean 260 –fldSD 73” parameter. Differentially expressed transcripts were analyzed using the high-throughput gene expression data analysis tool DIANE (https://diane.bpmp.inrae.fr/) (25). Differentially expressed transcripts were filtered by setting the log_2_ fold change (sgRNA OCT4/no sgRNA) to 0.58 and false discovery rate (FDR) to 0.05 as threshold values.

### 2.10 Functional annotation analysis

DAVID (https://www.david.ncifcrf.gov) (26) was used to identify the enriched molecular functions and pathways related to the genes of interest. *Q*-values (*P*-values adjusted for FDR) were calculated using the Benjamini–Hochberg method in DAVID.

Enrichr (http://amp.pharm.mssm.edu/Enrichr/) (27–29) was used to analyze the enriched molecular functions and pathways related to the differentially downregulated genes after OCT4-binding inhibition. “ENCODE and ChEA Consensus TFs from ChIP-X,” “TF Perturbations Followed by Expression,” and “ENCODE TF ChIP-seq 2015” were used as gene-set libraries. *Q*-values (*P*-values adjusted for FDR) were calculated using the Benjamini–Hochberg method in Enrichr.

### 2.11 Kaplan–Meier analysis-based prognosis classification of transcripts

Differentially downregulated genes detected in the RNA-seq analysis of CHP134 and IMR32 were input into the R2 Genomics Analysis and Visualization Platform (http://r2.amc.nl, Tumor Neuroblastoma - Kocak - 649 -custom - ag44kcwolf, GSE45547) for Kaplan–Meier analysis to extract a set of genes associated with a poor NB prognosis. For the type of survival, overall survival was selected. *Q*-values (*P*-values adjusted for FDR) were calculated using the Benjamini–Hochberg method in R2. We found 734 genes whose high expression was associated with a poor NB prognosis. The genes were classified into the “high is worse” group. We also identified 622 genes that were not associated with NB prognosis. The genes were classified into the “none” group. Subsequently, ORF dominance of the genes was calculated. Since a gene may potentially have multiple transcript isoforms, each with varying ORF dominance scores, the ORF dominance of a gene was determined by calculating the mean of the ORF dominance scores across all transcript isoforms.

### 2.12 Western blot analysis

The cells were lysed with RIPA buffer (50 mmol/L Tris‐HCl buffer (pH 7.6), 150 mmol/L NaCl, 1(w/v)% Nonidet P40 Substitute, 0.5(w/v)% sodium deoxycholate, protease inhibitor cocktail, and 0.1(w/v)% SDS; # 08714-04, Nacalai Tesque, Kyoto, Japan) and benzonase (Merck Millipore, Billerica, MA) and MgCl_2_ at final concentrations of 25 U/μL and 2 mM, respectively; incubated at 37°C for 1 h; and centrifuged at 10,000 × *g* for 10 min at 4°C. Thereafter, the supernatant was collected and denatured in SDS sample buffer (125 mM Tris-HCl, pH 6.8, 4% SDS, 10% sucrose, 0.01% BPB, and 10% 2-mercaptoethanol). Cellular proteins were resolved using sodium dodecyl sulfate-polyacrylamide gel electrophoresis before being electroblotted onto polyvinylidene fluoride membranes (#1704156, Bio-Rad Laboratories, Hercules, CA). The membranes were incubated with the following primary antibodies for 60 min at room temperature: anti-Cas9 (1:1000 dilution; #14697S, Cell Signaling Technology, Danvers, MA), anti-MDM2 (1:1000 dilution; OP46, Merck Millipore, Billerica, MA), anti-p53 (1:1000 dilution; #2524, Cell Signaling Technology, Danvers, MA), anti-caspase-2 (1:1000 dilution; sc-5292, Santa Cruz Biotechnology, Dallas, TX), anti-caspase 3 (1:1000 dilution; sc-7148, Santa Cruz Biotechnology, Dallas, TX), and anti-actin (1:1000 dilution; FUJIFILM Wako Pure Chemical Corporation, Osaka, Japan). The membranes were then incubated with horseradish peroxidase-conjugated secondary antibodies (anti-rabbit IgG at 1:5000 dilution or anti-mouse IgG at 1:5000 dilution; both from Cell Signaling Technology, Danvers, MA), and the bound proteins were visualized using a chemiluminescence-based detection kit (ImmunoStar Zeta; ImmunoStar LD, FUJIFILM Wako Pure Chemical Corporation, Osaka, Japan). Chemiluminescence was detected using ImageQuant™ LAS4000 (GE Healthcare, Chicago, IL).

### 2.13 Abby analysis

The MYCN protein levels were measured using a capillary electrophoretic-based immunoassay (the Abby instrument; ProteinSimple, San Jose, CA), according to the manufacturer’s protocol. Briefly, samples were combined with 0.1× sample diluent buffer and 5× fluorescent master mix denaturing buffer to acquire 0.8 µg/µL loading concentration. Subsequently, the samples were denatured for 5 min at 95 °C. The primary antibody used in the present study was MYCN (1:100 dilution; #9405, Cell Signaling Technology, Danvers, MA). The Abby measurement was performed using a 12–230 kDa separation module with 25-min separation at 375 V, 10-min blocking, 30-min primary antibody incubation, and 30-min secondary antibody incubation (DM-001, ProteinSimple, San Jose, CA). RePlex™ Module (RP-001, ProteinSimple, San Jose, CA) was used to detect total proteins. At the end of the run, the chemiluminescent signal was displayed as a virtual blot-like image and electropherogram based on the molecular weight using Compass (ProteinSimple, San Jose, CA).

### 2.14 Open reading frame dominance score analysis

The transcript sequences detected using long-read RNA-seq analysis were used to calculate ORF dominance, as previously described (30).

### 2.15 Statistical analysis

Statistical analysis software “R” was used for data analysis. Mann–Whitney *U*-test, Student’s *t*-test, and Kruskal–Wallis test were performed as appropriate. A *p*-value < 0.05 was considered statistically significant.

## 3 Results

### 3.1 CRISPR/dCas9 targeting transcription factor-binding sites at the *MYCN* locus reduced the proliferation in *MYCN*-amplified NB cells

**Figure 1:**
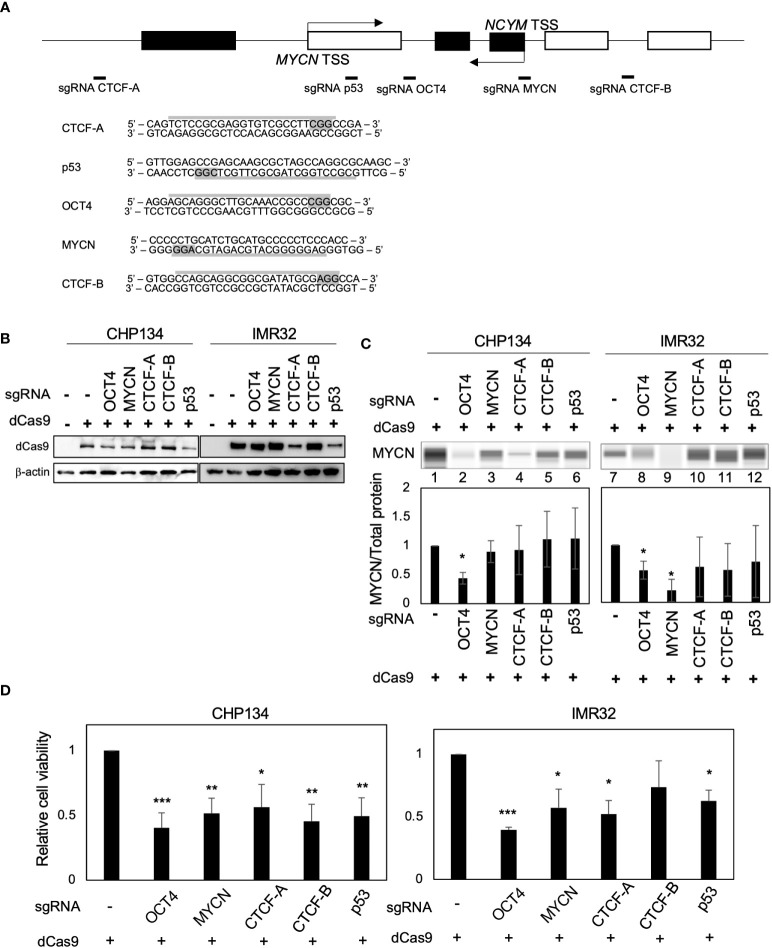
CRISPR/dCas9 targeting the *MYCN* locus reduces the proliferation of *MYCN*-amplified neuroblastoma cells. **(A)** A diagram of the MYCN/NCYM locus with the positions of targeting sgRNAs (Upper panel). The white and black boxes indicate the *MYCN* and *NCYM* regions, respectively. The lower panel highlights the targeting and PAM sequences in gray. TSS: transcription start site. **(B)** Western blotting of dCas9 protein in CHP134 and IMR32 cells. Twenty-four hours after CRISPR/dCas9 transfection, these cells were subjected to western blotting. *β-Actin* was used as a loading control. **(C)** Quantitative analysis was performed to measure the MYCN protein level. The MYCN protein level was assessed using the Abby instrument 72 h after transfection. The chemiluminescent signal is displayed as a virtual blot-like image (Upper panel). The quantified MYCN signal, normalized by the total protein level, is represented as a bar graph (Lower panel). Statistical significance (*: *p* < 0.05) was determined using the Student’s *t*-test, comparing the results with the those of the no sgRNA control. Error bars represent SEM of three independent experiments. **(D)** Ninety-six hours after CRISPR/dCas9 transfection, the proliferation of CHP134 and IMR32 was measured using the WST assay. *: *p* < 0.05; **: *p* < 0.01; ***: *p* < 0.001. Data were analyzed using Student’s *t-*test (compared with no sgRNA). Error bars represent SEM of six independent experiments.

Deactivated Cas9 (dCas9) disrupts the binding of transcription factors to specific sites (19). To inhibit transcription factor binding at the *MYCN* locus, we designed CRISPR guide RNAs against the MYCN-binding site (9, 10), OCT4-binding site (17), CTCF-binding site A (18), p53-binding site (20), and CTCF-binding site B (data from the UCSC Genome Browser) (**Figure 1A**). A previous study has demonstrated that CTCF binds to the upstream region of *MYCN* and promotes its transcription (18). However, using the UCSC Genome Browser, we discovered an additional CTCF-binding site located within the gene body of *MYCN* (**Figure S1**), whose function has not been investigated in previous study (18). Therefore, we designed CRISPR guide RNAs for both CTCF-binding sites. For convenience, we designated the CTCF-binding site upstream of *MYCN* as CTCF-A and the gene body region as CTCF-B (**Figures 1A** and **S1**). We transfected all-in-one CRISPR/dCas9-sgRNA vectors into CHP134 and IMR32 cells, both of which were *MYCN*-amplified NB cells (**Figure 1B**). MYCN expression in CHP134 and IMR32 cells decreased significantly when targeting the OCT4-binding site with dCas9 (**Figure 1C**, lane 2 and 8; representative raw data with loading control (total protein level) and MYCN signal are presented in **Figure S2**). The proliferation of CHP134 and IMR32 cells was significantly reduced by dCas9 targeting the OCT4-binding site, MYCN-binding site, CTCF-A site, and p53-binding site (**Figure 1D**). Among these targets, dCas9 targeting the OCT4-binding site was most significantly decreased in the proliferation of both *MYCN*-amplified NBs (CHP134 and IMR32) (**Figure 1D**). In contrast, the proliferation of the *MYCN*-nonamplified NB cell line SK-N-AS cell line, exhibiting a lower expression of *OCT4 (POU5F1)* mRNA relative to other NB cell lines (**Figure S3A**), was not affected by dCas9 targeting the OCT4-binding site (**Figure S3B**).

### 3.2 Inhibition of OCT4 binding at the *MYCN* locus suppresses *MYCN* mRNA and MYCN activity

**Figure 2:**
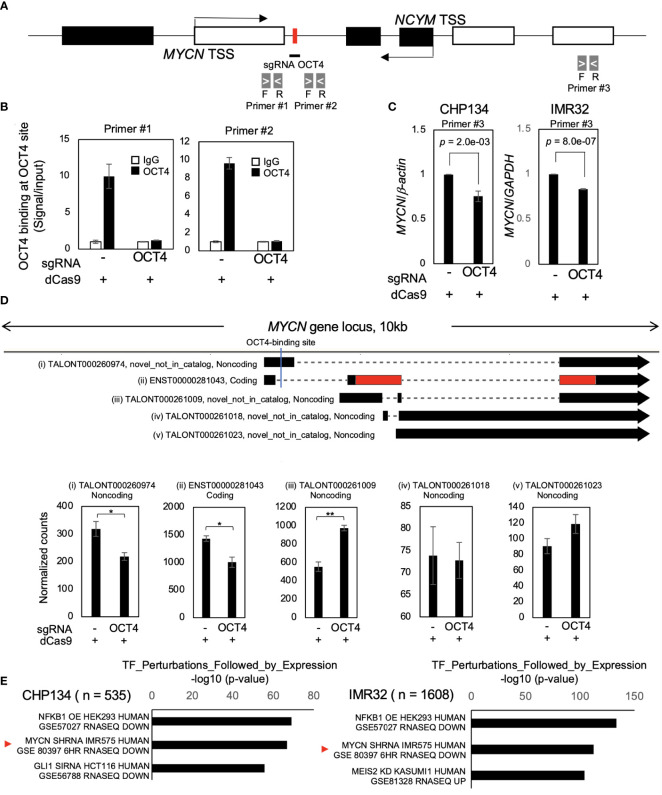
Inhibition of OCT4 binding at the *MYCN* locus suppresses *MYCN* mRNA and MYCN activity. **(A)** Schematic depiction of the MYCN/NCYM locus with the location of the primers used in the CUT&RUN assay and quantitative real-time reverse transcription-polymerase chain reaction (qRT-PCR). The OCT4-binding site is indicated with a red box. The white and black boxes indicate the *MYCN* and *NCYM* regions, respectively. The primers employed in the CUT&RUN assay are denoted as primers #1 and #2, whereas that used in qRT-PCR is denoted as primer #3. **(B)** OCT4 binding at the *MYCN* locus was inhibited using CRISPR/dCas9. Twenty-four hours after the transfection of CRISPR/dCas9 targeting the OCT4-binding site, CHP134 cells were subjected to the CUT&RUN assay. Genomic DNA was amplified via qRT-PCR using primers #1and #2 in **(A)**. The signals were normalized by input signals. IgG was used as an isotype control. Error bars represent SEM of three technical replicates. The data presented are representative of the experiment. **(C)** qRT-PCR analyses of *MYCN* in CRISPR/dCas9-transfected CHP134 and IMR32 cells. One day after transfection, *MYCN* mRNA expression levels were measured via qRT-PCR using primer #3 in **(A)** with *β-actin* or *GAPDH* as an internal control. Data were analyzed using Student’s *t-*test. Error bars represent SEM of three independent experiments. **(D)** A diagram of transcripts detected at the *MYCN* locus (Upper panel). Black regions indicate *MYCN* transcripts. Red regions indicate coding sequences (CDS). Novel_not_in_catalog means a novel transcript not in the reference produced by a novel splice site. An OCT4-binding site is indicated by a blue line in the diagram. The lower panel demonstrates normalized expression counts (TPM) of *MYCN* transcripts from short-read RNA-seq analysis in CHP134 cells. Error bars represent SEM of three independent experiments. Data were analyzed using the Student’s *t-*test. *: *p* < 0.05; **: *p* < 0.01. **(E)** Differentially downregulated genes after inhibition of OCT4 binding at the *MYCN* locus were enriched in MYCN*-*target genes. Enrichr analysis (http://amp.pharm.mssm.edu/Enrichr/) summary of enriched transcription factor-target genes.

As cell proliferation was significantly reduced by dCas9 targeting the OCT4-binding site in both *MYCN*-amplified NB cell lines (**Figure 1D**), we investigated the effect of inhibition of the OCT4-binding site on MYCN activity. Twenty-four hours after dCas9 transfection, CRISPR/dCas9 inhibited OCT4 binding at the *MYCN* locus (**Figures 2A, B**). At this time, the levels of *OCT4* mRNA remained unchanged (**Figure S4A**), indicating that the decrease in OCT4 binding was not dependent on the expression levels of OCT4. We investigated the potential binding of alternative transcription factors to the OCT4-binding site in *MYCN* locus using the UCSC Genome Browser. However, our findings show that there are no alternative transcription factors found in the same location as the OCT4-binding site in NB (**Figure S4B**). This indicates that there is no evidence supporting the idea that dCas9 inhibits transcription factors other than OCT4. Additionally, we conducted an investigation into an alternative potential effect of dCas9, specifically its role in interference with transcription elongation (31). We examined the recruitment of RNA polymerase II (RNA pol II) in the proximity of the OCT4-binding site within the *MYCN* locus using the CUT&RUN assay. The results revealed that there was no alteration in both the recruitment of RNA pol II and its phosphorylation status (**Figures S4C and S4D**), indicating that dCas9 does not interfere with the process of transcription elongation in the proximity of the OCT4-binding site.

The inhibition of OCT4 binding at the *MYCN* locus suppressed the expression of *MYCN* mRNA compared to control (dCas9 without sgRNA) (**Figure 2C**). A similar trend of *MYCN* mRNA reduction was also observed on transfection of dCas9 targeting MYCN-binding site; however, it did not reach statistical significance (**Figure S5**). Notably, the reduction in *MYCN* expression resulting from OCT4 inhibition was modest (**Figure 2C**). The finding seems to contradict the substantial decrease in MYCN protein expression levels observed in **Figure 1C**. To precisely identify and characterize the transcriptomic changes resulting from OCT4 binding inhibition, we performed short-read RNA-seq combined with long-read RNA-seq of CHP134 and IMR32 cells 24 h after dCas9 transfection. We have listed the detected transcripts and their expression levels in **Table S1** (can be accessed on FigShare; https://doi.org/10.6084/m9.figshare.24543067.v1). Through this analysis, we detected 17,601 annotated transcripts (transcript ID starts with ENST~) and 70,753 unannotated transcripts (transcript ID starts with TALONT~) in the combined CHP134 and IMR32 cell samples. Notably, the number of unannotated transcripts was approximately four times higher than the number of annotated transcripts. In the *MYCN* locus, we detected one annotated transcript (**Figure 2D** (ii)) and four unannotated transcripts (**Figure 2D** (i), (iii), (iv), and (v)) using long-read RNA-seq analysis. Notably, the expression of ENST00000281043 (**Figure 2D** (ii)), which encodes the MYCN protein, was suppressed. The result is consistent with the downregulation of MYCN expression in **Figure 1C**. In contrast, noncoding transcripts of *MYCN* with the highest expression levels showed increased expression (TALONT000261009, **Figure 2D** (iii)). Because the primer set #3 detected both the coding and noncoding transcripts, the reduction of *MYCN* mRNA appear to be weak in RT-qPCR (**Figure 2C**). The results suggest that OCT4 regulates promoter usage of *MYCN* gene, and its binding inhibition promotes transcription from the internal promoter (**Figure 2D**), resulting in reduction of MYCN protein level (**Figure 1C**). In the *MYCNOS (NCYM)* locus, we detected two annotated transcripts (**Figure S6A** (viii) and (ix)) and two unannotated transcripts (**Figure S6A** (vi) and (vii)) using long-read RNA-seq analysis. Among these transcripts, we observed an upregulation in the expression levels of TALONT000260926 (**Figure S6B** (vii)) and ENST00000419083 (**Figure S6B** (viii)), both of which have unknown functions. The data provide evidence for the presence of previously unannotated transcripts transcribed from the *MYCN*/*NCYM* locus in NB. Moreover, OCT4 regulates the expression of particular isoforms within the *MYCN*/*NCYM* locus, including the protein-coding isoform of *MYCN*.

In order to examine the potential impact of reducing MYCN expression levels on the downstream pathway of MYCN, we conducted Enrichr analysis (http://amp.pharm.mssm.edu/Enrichr/) (27–29). Enrichr analysis revealed that differentially downregulated genes were enriched in MYCN-target genes (GSE80397: downregulated gene set after *MYCN* knockdown in IMR575) (**Figure 2E**) and MYC/MAX-target genes (**Table S2**). On the contrary, in the Enrichr analysis of three independent gene-set libraries (ENCODE and ChEA Consensus TFs from ChIP-X, TF Perturbations Followed by Expression, and ENCODE TF ChIP-seq 2015), enrichment of OCT4-target genes was not observed (**Table S2**), suggesting no off-target effects of CRISPR/dCas9 on the expression of other OCT4-target genes. These findings indicate that CRISPR/dCas9 specifically inhibited OCT4 binding at the *MYCN* locus and suppressed MYCN activity in *MYCN*-amplified NB.

### 3.3 Inhibition of OCT4 binding at the *MYCN* locus induces NB cell death accompanied by downregulation of transcripts with high-ORF dominance

**Figure 3:**
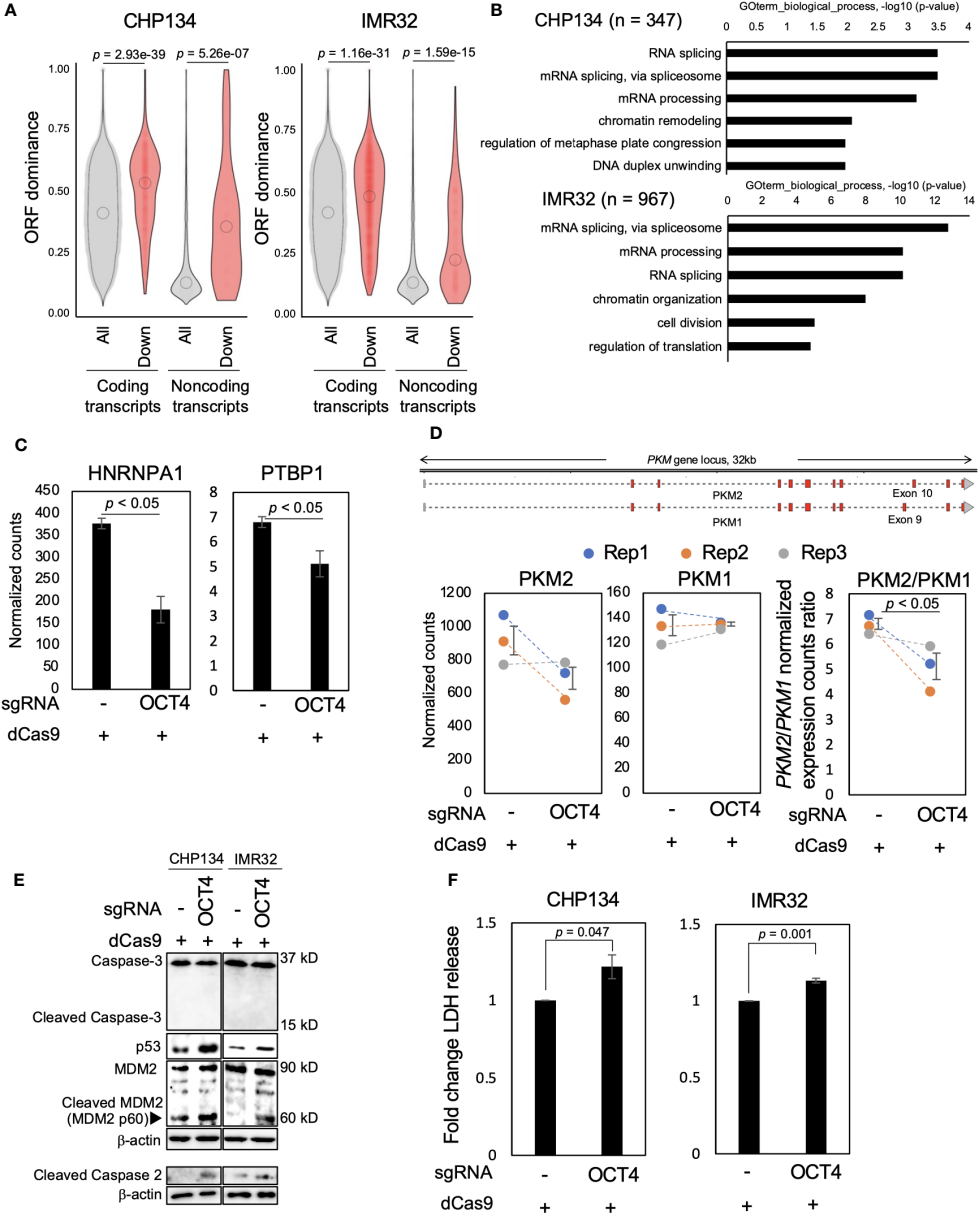
Inhibition of OCT4 binding at the *MYCN* locus induces neuroblastoma (NB) cell death accompanied by downregulation of transcripts with high-open reading frame (ORF) dominance. **(A)** Differentially downregulated transcripts were associated with high-ORF dominance in CHP134 (left) and IMR32 cells (right). The number of samples was as follows: coding transcripts (CHP134: all, n = 51,400; down, n = 610, IMR32: all, n = 53,245; down, n = 2,047). Noncoding transcripts (CHP134: all, n = 3,464; down, n = 29, IMR32: all, n = 3,601; down, n = 144). The summary of the data is shown as a violin plot reflecting the data distribution and an open circle indicating the median of the data. *P*-values were calculated using Mann–Whitney *U*-test. **(B)** Gene Ontology (GO) analysis of differentially downregulated transcripts with high-ORF dominance (ORF dominance > 0.5). **(C)** Normalized expression counts (TPM) for *HNRNPA1* and *PTBP1* transcripts from short-read RNA-seq analysis in CHP134 cells. Error bars represent SEM of three independent experiments. Data were analyzed using the Student’s *t*-test. **(D)** The CRISPR/dCas9 targeting of the OCT4-binding site led to a reduction in the *PKM2*/*PKM1* ratio. Upper panel: a diagram of transcripts detected using the long-read RNA-seq analysis at the *PKM* locus. Red regions indicate coding sequences (CDS). Lower panel: Normalized expression counts (TPM) for *PKM2* and *PKM1* isoforms and *PKM2/PKM1* normalized expression count ratio from short-read RNA-seq analysis in CHP134 cells. Blue, orange, and gray dots correspond to biological replicates 1, 2, and 3, respectively. Error bars represent SEM of three independent experiments. Data were analyzed using the Student’s *t*-test. **(E)** Western blotting of p53, MDM2, caspase-2, and caspase-3 in dCas9-transfected NB cells. Seventy-two hours after transfection, the cells were subjected to western blotting. β-actin was used as the loading control. **(F)** CRISPR/dCas9 targeting the OCT4-binding site induced NB cell death. Ninety-six hours after transfection of CRISPR/dCas9, activity of lactate dehydrogenase (LDH) released from cells was measured using the cytotoxicity LDH assay. Data were analyzed using the Student’s *t*-test. Error bars represent SEM of three independent experiments.

We examined how the reduced MYCN activity altered the NB transcriptome. In our previous study, we developed the ORF dominance score, which is defined as the fraction of the longest ORF in the sum of all putative ORF lengths within a transcript sequence (30). This score correlates with translation efficiency of coding transcripts and noncoding RNAs (30). Our previous *in silico*-based analysis suggested that noncoding transcripts with high-ORF dominance are associated with downstream genes of MYCN in humans (30). Therefore, we investigated whether MYCN functions as a regulator of transcripts with high-ORF dominance in NB. We calculated ORF dominance scores of differentially downregulated transcripts using long-read RNA-seq analysis (**Table S3**). The differentially downregulated transcripts had significantly higher ORF dominance than all transcripts, and this trend was observed for both coding and noncoding RNAs (**Figure 3A**). Additionally, isoform expression analysis from short-read RNA-seq showed similar results, revealing that the differentially downregulated transcripts had significantly higher ORF dominance in both coding and noncoding transcripts (**Figure S7**). These findings indicate that MYCN maintains the expression of transcripts with high-ORF dominance in NB. Given that ORF dominance correlates with the translation efficiency of transcripts (30), our results suggest that MYCN maintains the expression of transcripts with high translation efficiency in NB.

MYCN has been reported to globally regulate transcription (32) and splicing (33); however, how this transcription and splicing regulations link to the proteome remains elusive. Since ORF dominance correlates with translation efficiency of transcripts (30), our results suggest that these MYCN-induced RNA isoform changes are directional, with increasing potential for efficient translation. Subsequently, we extracted transcripts with high-ORF dominance (ORF dominance > 0.5) from the differentially downregulated transcripts and performed Gene Ontology (GO) analysis using DAVID. Transcripts with high-ORF dominance were associated with the GO terms “mRNA processing,” “mRNA splicing via spliceosome,” and “RNA splicing” (**Figure 3B** and **Table S4**). Notably, the genes encoding the splicing factors *HNRNPA1* and *PTBP1* are the targets of MYCN, and a decrease in MYCN activity induces the downregulation of *HNRNPA1* and *PTBP1* expression and suppresses the proliferation of *MYCN*-amplified NB cells (33). Consistent with this previous report, the expression of *HNRNPA1* and *PTBP1*, the target genes of MYCN, was downregulated after OCT4-binding inhibition in this study (**Figure 3C**). HNRNPA1 and PTBP1 regulate the alternative splicing of the pyruvate kinase gene (*PKM*) and facilitate the switch from the canonical isoform *PKM1* to the cancer-related isoform *PKM2* (33, 34). The knockdown of *PTBP1*, *HNRNPA1*, and their downstream target *PKM2* represses the proliferation of *MYCN*-amplified NB (33). Similarly, the *PKM2*/*PKM1* ratio was considerably decreased by OCT4-binding inhibition in this study (**Figure 3D**), suggesting that the splicing switch from *PKM1* to *PKM2* underlies the mechanism of inhibition of NB proliferation after transfection of CRISPR/dCas9 targeting the OCT4-binding site.

We examined the expression of cell death-related proteins using western blotting to gain insights into the mechanism of inhibition of NB proliferation. In our previous study, we found that blocking the p53-binding site at the *MYCN* locus using CRISPR/dCas9 results in the cleavage of caspase-2 and MDM2 and induction of p53 expression (20), which is associated with the p53–MDM2–caspase-2 positive feedback loop (35). Consistent with this report, cleavage of caspase-2 and MDM2, but not caspase-3, and induction of p53 expression were observed in *MYCN*-amplified NB cells at 72 h after transfection of CRISPR/dCas9 (**Figure 3E**). To evaluate cytotoxicity, the activity of lactate dehydrogenase (LDH) released from cells was measured using a cytotoxicity LDH assay at 96 h after transfection of CRISPR/dCas9. The LDH assay is a method commonly used to evaluate cell damage. LDH is a stable cytoplasmic enzyme that is found in all cells and is released rapidly into the cell culture supernatant when the plasma membrane is damaged (36). The LDH activity was enhanced by CRISPR/dCas9 targeting the OCT4-binding site in CHP134 and IMR32 cells (**Figure 3F**). This result suggests that inhibition of OCT4 binding at the *MYCN* locus induces apoptosis in NB cells via the activation of the p53–MDM2–caspase-2 positive feedback loop.

### 3.4 Genes associated with poor NB prognosis encode high-ORF dominance transcripts

**Figure 4:**
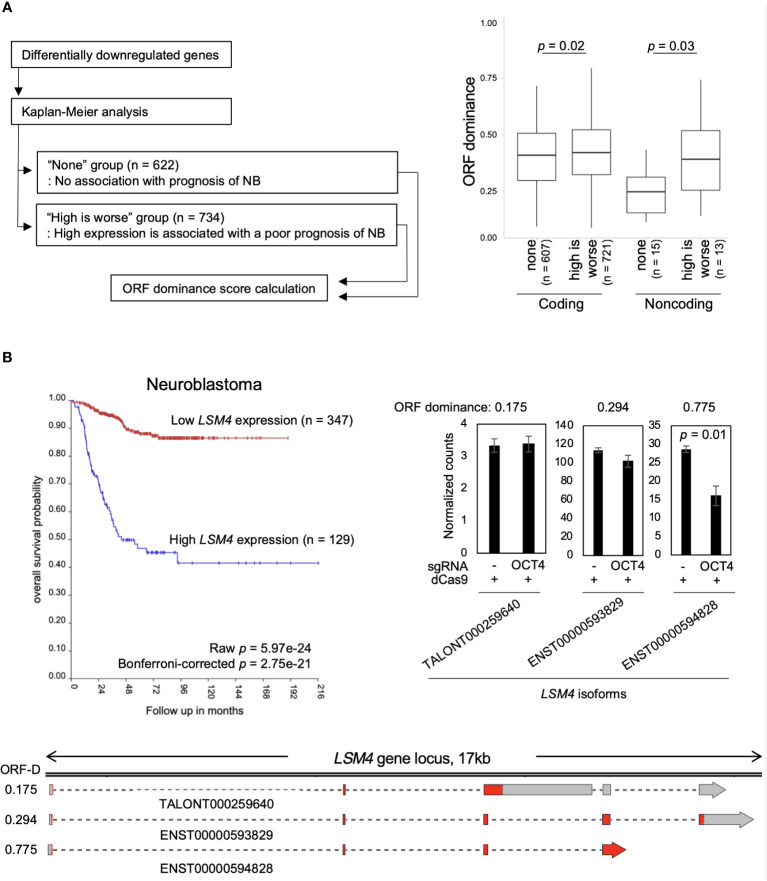
Genes associated with the poor neuroblastoma (NB) prognosis encode high-ORF dominance transcripts. **(A)** The “high is worse” transcripts (coding, n = 721; noncoding, n = 13) showed higher ORF dominance than the “none” transcripts (coding, n = 607; noncoding, n = 15). The “high is worse” group contains transcripts whose high expression is associated with a poor NB prognosis. The “none” group transcripts are not associated with NB prognosis. The summary of the data is shown as a boxplot, with the box indicating the IQR, the whiskers showing the range of values that are within 1.5*IQR and a horizontal line indicating the median. *P*-values were calculated using the Mann–Whitney *U*-test. **(B)** High expression of *LSM4* was associated with a poor NB prognosis (Bonferroni-corrected *p* = 2.75e-21) (Upper left panel). The upper right panel displays normalized expression counts (TPM) of *LSM4* transcripts from short-read RNA-seq analysis in CHP134 cells. Error bars represent SEM of three independent experiments. Data were analyzed using the Student’s *t-*test. The lower panel displays a diagram of transcripts detected at the *LSM4* locus in CHP134. Red regions indicate coding sequences (CDS). ORF-D: ORF dominance.

Finally, the relationship between NB prognosis and ORF dominance was investigated. For this analysis, the R2 Genomics Analysis and Visualization Platform was used. Differentially downregulated genes were input into the Kaplan−Meier analysis of the R2 database to extract a set of genes associated with poor NB prognosis. We found 734 genes whose high expression is associated with a poor NB prognosis. The genes were classified into the “high is worse” group (**Table S5**). In addition, 622 genes that were not associated with NB prognosis were identified, which were classified into the “none” group (**Table S5**). Afterward, ORF dominance of the genes was calculated. Our analysis revealed that coding transcripts in the ‘high is worse’ group exhibited slightly higher but statistically significant ORF dominance than those in the ‘none’ group (**Figure 4A**). Additionally, in the noncoding transcripts, the ‘high is worse’ group showed higher ORF dominance than those in the ‘none’ group (**Figure 4A**). Here, an example of a gene from “high is worse” group is presented. *LSM4*, a MYCN-target gene (37), encodes a member of the LSm family of RNA-binding proteins. LSM4 plays a role in pre-mRNA splicing as a component of the U4/U6-U5 tri-snRNP complex (38). High *LSM4* expression is significantly associated with a poor NB prognosis (**Figure 4B**). After inhibiting the OCT4 binding at the *MYCN* locus, only the *LSM4* isoform with high-ORF dominance (ENST00000594828, ORF dominance = 0.775) was downregulated, whereas other isoforms remained unchanged (**Figure 4B**). The results indicate that genes associated with the poor NB prognosis encode high-ORF dominance transcripts, and OCT4 binding inhibition at the *MYCN* locus suppresses these high-ORF dominance transcripts in NB. Because ORF dominance correlates with the translation efficiency of transcripts (30), our results suggest that MYCN maintains the expression of transcripts with high translation efficiency, thereby contributing to a poor prognosis in NB.

## 4 Discussion

Conventional studies on transcriptional regulation of *MYCN* have largely relied on knockdown/knockout or overexpression of transcription factors, and such methods alter the expression levels of transcription factors, resulting in activation/suppression of downstream target genes other than *MYCN*. Therefore, subsequent alterations in *MYCN* expression by such methods include indirect effects of pathway activation/suppression other than direct effect of transcription factor binding on *MYCN* locus. The CRISPR/dCas9 system was employed to elucidate the significance of transcription factor binding on *MYCN* locus, and found that the inhibition of OCT4 binding was found to be critical for *MYCN* expression in *MYCN*-amplified NB. In our previous study, high *OCT4* mRNA expression was found to be associated with a poor prognosis of *MYCN*-amplified NBs, but not in *MYCN*-non-amplified NBs (17). Consistent with the finding, in the present study, OCT4-binding inhibition in the intron 1 region of *MYCN* decreased the proliferation of *MYCN*-amplified NB cells (CHP134 and IMR32) but not that of *MYCN*-non-amplified NB cells (SK-N-AS), further suggesting that the human-specific OCT4–MYCN network is specifically required for the survival of *MYCN*-amplified NB. Furthermore, long-read sequencing analyses revealed that OCT4 binding on the *MYCN* locus regulates promoter usage in CHP134 and inhibition of the binding resulted in stimulation of transcription of noncoding transcript of *MYCN* from internal promoter. Therefore, combination of the CRISPR/dCas9 system and long-read RNA sequencing clarified the novel mechanism of transcriptional regulation of *MYCN* that could not be revealed by conventional methods.

Inhibition of OCT4 binding at the *MYCN* locus suppressed *MYCN* and its downstream genes, including *HNRNPA1* and *PTBP1*, which are splicing factors. The reduction of *HNRNPA1* and *PTBP1* subsequently decreased splicing activity, leading to a decrease in the *PKM2*/*PKM1* ratio and activation of caspase-2. A previous study by Zhang et al. (33) reported that knockdown of PKM2 suppresses cell proliferation in *MYCN*-amplified NB cells (IMR5), but not in *MYCN*-non-amplified NB cells (SK-N-AS), suggesting a *MYCN*-amplified NB-dependent function for PKM2. Our observation that CRISPR/dCas9 targeting of the OCT4 binding site suppresses cell proliferation specifically in *MYCN*-amplified NB is therefore consistent with this report. However, the link between PKM2 and caspase-2 remains unclear. One possible explanation is that PKM2 interacts with the CDK1-cyclinB complex to facilitate cell cycle progression in gliomas, and knockdown of PKM2 decreases CDK1 kinase activity (39). Reduced CDK1 activity decreases the inhibitory phosphorylation level of the S340 residue of caspase-2, thereby leading to caspase-2 activation (40). Thus, suppressed *PKM2* expression may activate caspase-2 through the reduction of CDK1-cyclin B kinase activity.

In addition to the OCT4–MYCN network, the inhibition of super enhancer is an interesting approach for targeting MYCN expression in NB. NB is characterized by a core regulatory circuitry (CRC) comprising transcription factors such as PHOX2B, HAND2, and GATA3 that regulate super enhancer (41, 42). The transcription factors form a network with MYCN (43) and are essential for maintaining the cell state in *MYCN*-amplified NB (44). Because blocking of OCT4 or MYCN reduces MYCN expression, simultaneous blocking of OCT4 or MYCN with this CRC, with a focus on targeting transcription factors including HAND2, PHOX2B, and GATA3, may be an effective therapeutic approach for NB.

Previously, we developed the ORF dominance score, defined as the fraction of the longest ORF in the sum of all putative ORF lengths (30). An *in silico*-based analysis suggested that noncoding transcripts with high-ORF dominance are associated with the downstream gene of MYCN in humans (30). However, whether MYCN regulates the expression of transcripts with high-ORF dominance has not yet been experimentally investigated. In this study, we investigated the effect of MYCN activity on the expression of transcripts with high-ORF dominance in *MYCN*-amplified NB cells. Our findings demonstrate that a reduction in MYCN activity led to a decrease in the expression of both coding and noncoding transcripts with high ORF dominance. Because ORF dominance correlates with the translation efficiency of transcripts (30), our results suggest that MYCN maintains the expression of transcripts with high translation efficiency. Importantly, this study identifies MYCN as the first experimentally validated regulator of ORF dominance. However, the mechanism by which MYCN regulates ORF dominance remains unclear. Currently, two potential scenarios are under consideration: (i) MYCN directly transcribes transcripts with high-ORF dominance, or (ii) MYCN maintains expression of transcripts with high-ORF dominance through splicing. In the present study, we observed downregulation of MYCN and its target genes following OCT4 inhibition. As these differentially downregulated transcripts exhibited high ORF dominance, it is plausible that MYCN directly transcribes transcripts with high ORF dominance. However, it has been reported that MYCN transcribes splicing factors (33) and, in the present study, splicing factors *HNRNPA1* and *PTBP1*, which are MYCN-target genes, were downregulated after inhibition of OCT4 binding. As alternative splicing potentially alters the ORF dominance of transcripts, MYCN may maintain the expression of transcripts with high ORF dominance through splicing. Further research is required to elucidate the precise mechanism by which MYCN regulates ORF dominance. Moreover, we found that genes associated with the poor prognosis of NB encode high-ORF dominance transcripts, indicating that ORF dominance may serve as a novel prognostic marker in NB. Coding transcripts in the ‘high is worse’ group exhibited significantly higher ORF dominance than those in the ‘none’ group, although this difference was slight. However, the ORF dominance data obtained in this study were based on transcript sequences from cell lines (CHP134 and IMR32), which may not provide the most accurate calculation of ORF dominance. Long-read RNA sequencing data from the large number of clinical samples of NB, which are required for the accurate calculation of ORF dominance, are not currently available. Hence, future studies should investigate whether the ORF dominance score can serve as a prognostic marker in NB using patient-derived transcript data.

## Data availability statement

The datasets presented in this study can be found in online repositories. The names of the repository/repositories and accession number(s) can be found in the article/**Supplementary Material**.

## Ethics statement

Ethical approval was not required for the studies on humans in accordance with the local legislation and institutional requirements because only commercially available established cell lines were used.

## Author contributions

KN, HK, TM, KK, YHa, TSa, TY, and YS performed the experiments and acquired and analyzed the data. KN, KK, YHa, YHi, and YS wrote the manuscript. KN, TSe, YHi, and YS acquired the funds. YS designed and supervised the study. All authors contributed to the article and approved the submitted version.
