# Supplementary material for: Inhibition of OCT4 binding at the MYCN locus induces neuroblastoma cell death accompanied by downregulation of transcripts with high-open reading frame dominance
Source: Front Oncol. 2024 Feb 8;14:1237378. doi: 10.3389/fonc.2024.1237378 (PMC10882222; doi:10.3389/fonc.2024.1237378)
Supplement: Supplementary file 1 [file DataSheet_1.zip › Supplementary text including Supplementary Table 1.docx]

Supplementary Material

Inhibition of OCT4 Binding at the *MYCN* Locus Induces Neuroblastoma Cell Death Accompanied by Downregulation of Transcripts with High-Open Reading Frame Dominance

Kazuma Nakatani, Hiroyuki Kogashi, Takanori Miyamoto, Taiki Setoguchi, Tetsushi Sakuma, Kazuto Kugou, Yoshinori Hasegawa, Takashi Yamamoto, Yoshitaka Hippo, Yusuke Suenaga^*^

*** Correspondence:** Yusuke Suenaga: ysuenaga@chiba-cc.jp

# Supplementary Figures and Tables

**Figure S1.** CCCTC-binding factor (CTCF) enrichment at the *MYCN* locus.
UCSC Genome Browser showing the enrichment of CTCF in the upstream region of the transcription start site (TSS) (CTCF-A) and gene body (CTCF-B) of *MYCN* in GSE115862 neuroblastoma data set.

**Figure S2.** Representative raw data from the quantification of MYCN protein levels in CHP134 and IMR32 after 72 h of dCas9 transfection.
 Electropherograms were generated based on molecular weights. The total protein peak area is depicted by the gray area surrounded by a blue line, while the MYCN protein peak area is represented by the green area.

**Figure S3.** Cell proliferation was unchanged by deactivated cas9 (dCas9) targeting the OCT4-binding site in *MYCN* non-amplified neuroblastoma cell line (SK-N-AS).
(A) Heatmap representation of *OCT4 (POU5F1)* expression levels in 9 neuroblastoma cell lines, measured in transcripts per million (TPM). The data source is the Cancer Cell Line Encyclopedia (45, 46). (B) Ninety-six hours after CRISPR/dCas9 transfection, the proliferation of SK-N-AS cells was measured using the WST assay. Data were analyzed using student’s *t-*test (compared with no sgRNA). Error bars represent SEM (n = 3).

**Figure S4.** dCas9 targeting the OCT4 binding site did not affect alternative transcription factors and the RNA polymerase II traffic.
(A) *OCT4* mRNA expression was unchanged by deactivated cas9 (dCas9) targeting the OCT4-binding site in CHP134. qRT-PCR analyses of *MYCN* were employed in CRISPR/dCas9-transfected CHP134 cells. 24 h after transfection, *OCT4* mRNA expression levels were measured using qRT-PCR with *β-actin* as an internal control. Data were analyzed using Student’s *t-*test. Error bars represent the standard deviation (SD) of three independent experiments. (B) UCSC Genome Browser showing the enrichment of transcription factors in the *MYCN* locus in neuroblastoma data set (GSE115862). Red line indicates OCT4-binding site. (C) Schematic depiction of the *MYCN*/*NCYM* locus with the location of the primers used in the CUT&RUN assay and quantitative real-time reverse transcription-polymerase chain reaction (qRT-PCR). The OCT4-binding site is indicated with a blue line. The white and black boxes indicate the *MYCN* and *NCYM* regions, respectively. TSS: transcription start site. (D) RNA polymeraseⅡ (PolⅡ) dynamics at the *MYCN* locus. Twenty-four hours after the transfection of CRISPR/dCas9 targeting the OCT4-binding site, CHP134 cells were subjected to the CUT&RUN assay using anti-RNA PolⅡ antibody, anti-RNA PolⅡ C-terminal domain (CTD) phospho Ser2 (Pol Ⅱ pSer2) antibody, and anti-RNA PolⅡ CTD phospho Ser5 (Pol Ⅱ pSer5) antibody. Genomic DNA was amplified via qRT-PCR using primers #1, #2, #3, #4, #5, #6, and #7 in Figure S4C. The signals were normalized by IgG signals and input signals. Error bars represent SEM of three biological replicates. The “pSer2/Pol II” parameter denotes the ratio obtained from the signals of Pol Ⅱ pSer2 and RNA Pol II (Upper right panel). The “pSer5/Pol II” parameter denotes the ratio obtained from the signals of Pol Ⅱ pSer5 and RNA Pol II (Lower right panel). Data were analyzed using student’s *t-*test (compared with no sgRNA). Error bars represent SEM (n = 3).

**Figure S5.** *MYCN* mRNA expression was unchanged by deactivated cas9 (dCas9) targeting the MYCN-binding site in CHP134.
qRT-PCR analyses of *MYCN* were employed in CRISPR/dCas9-transfected CHP134 cells. One day after transfection, *MYCN* mRNA expression levels were measured using qRT-PCR using primer #3 in Figure 2A with *β-actin* as an internal control. Data were analyzed using Student’s *t-*test. Error bars represent SEM of three independent experiments.

**Figure S6.** *MYCNOS* (*NCYM*) transcripts detected using long-read RNA-seq analysis.
(A) A diagram of transcripts detected at the *MYCN*/*NCYM* locus. Black and gray indicate *MYCN* and *MYCNOS* (*NCYM*) transcripts, respectively. Red regions indicate coding sequences (CDS). Novel_not_in_catalog means a novel transcript not in the reference produced by a novel splice site. Novel_in_catalog means a novel transcript not in the reference produced by a known splice site. (B) Normalized expression counts (TPM) for *MYCNOS* (*NCYM*) transcripts from short-read RNA-seq analysis in CHP134 cells. Error bars represent SEM of three independent experiments. Data were analyzed using Student’s *t-*test. *: *p* < 0.05; **: *p* < 0.01; ***: *p* < 0.001.

**Figure S7.** Differentially downregulated transcripts had a high-open reading frame (ORF) dominance score (short-read RNA-seq analysis).
Differentially downregulated transcripts were associated with high-ORF dominance in CHP134 (left) and IMR32 (right) cells. The number of samples was as follows: coding transcripts (CHP134; all, n = 141,246, up, n =1,047, down, n = 2,286, IMR32; all, n = 142,570, up, n = 5,618, down, n = 4,255). Noncoding transcripts (CHP134; all, n = 18,328, up, n = 325, down, n = 70, IMR32; all, n = 19,691, up, n = 697, down, n = 173). A summary of data is shown as a boxplot, with the box indicating the IQR, whiskers showing the range of values that were within 1.5*IQR, and horizontal line indicating the median. *P*-values were calculated using Kruskal–Wallis test. *: *p* < 1.0e-03; **: *p* < 1.0e-06; ***: *p* < 1.0e-19

**Table S1.**The gene information including open reading frame (ORF) dominance score and normalized expression counts of each transcript (n=3 per condition). (can be accessed on FigShare; https://doi.org/10.6084/m9.figshare.24543067.v1)

**Table S2.**Enrichr analysis of differentially downregulated genes after OCT4-binding inhibition in CHP134 and IMR32.

**Table S3.**The ORF dominance of differentially downregulated transcripts.

**Table S4.**Gene Ontology (GO) analysis of differentially downregulated transcripts with high-ORF dominance (ORF dominance > 0.5).

**Table S5.**List of genes analyzed using the Kaplan-Meier method.

45. Barretina J, Caponigro G, Stransky N, Venkatesan K, Margolin AA, Kim S, et al. The Cancer Cell Line Encyclopedia enables predictive modelling of anticancer drug sensitivity. Nature (2012) 483:603–7. doi: [10.1038/nature11003](https://doi.org/10.1038/nature11003).

46. Ghandi M, Huang FW, Jané-Valbuena J, Kryukov GV, Lo CC, McDonald ER, et al. Next-generation characterization of the Cancer Cell Line Encyclopedia. Nature (2019) 569:503–8. doi: [10.1038/s41586-019-1186-3](https://doi.org/10.1038/s41586-019-1186-3).
