# Supplementary figures and images for: Inhibition of OCT4 binding at the MYCN locus induces neuroblastoma cell death accompanied by downregulation of transcripts with high-open reading frame dominance
Source: Front Oncol. 2024 Feb 8;14:1237378. doi: 10.3389/fonc.2024.1237378 (PMC10882222; doi:10.3389/fonc.2024.1237378)

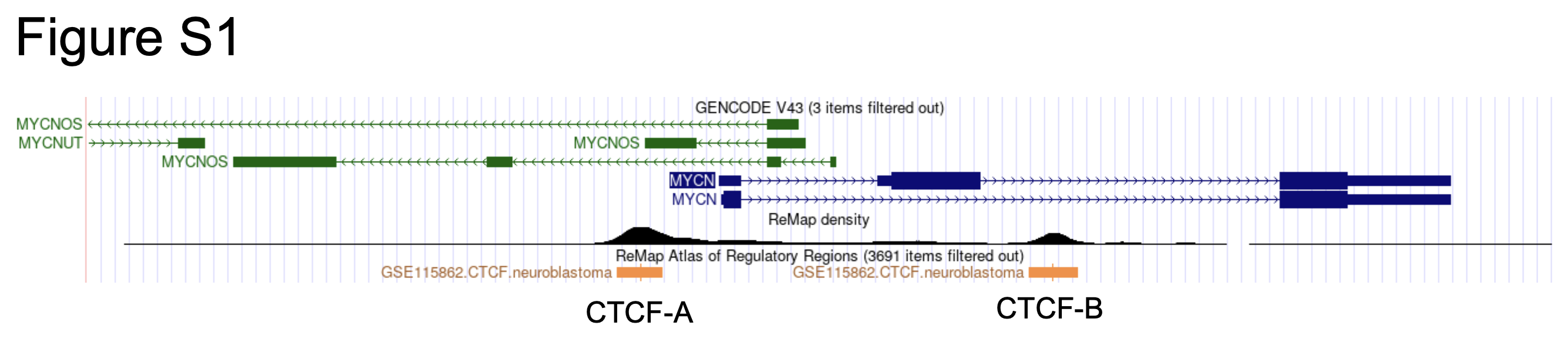

Supplement: Supplementary file 2 [file Image_1.tiff]

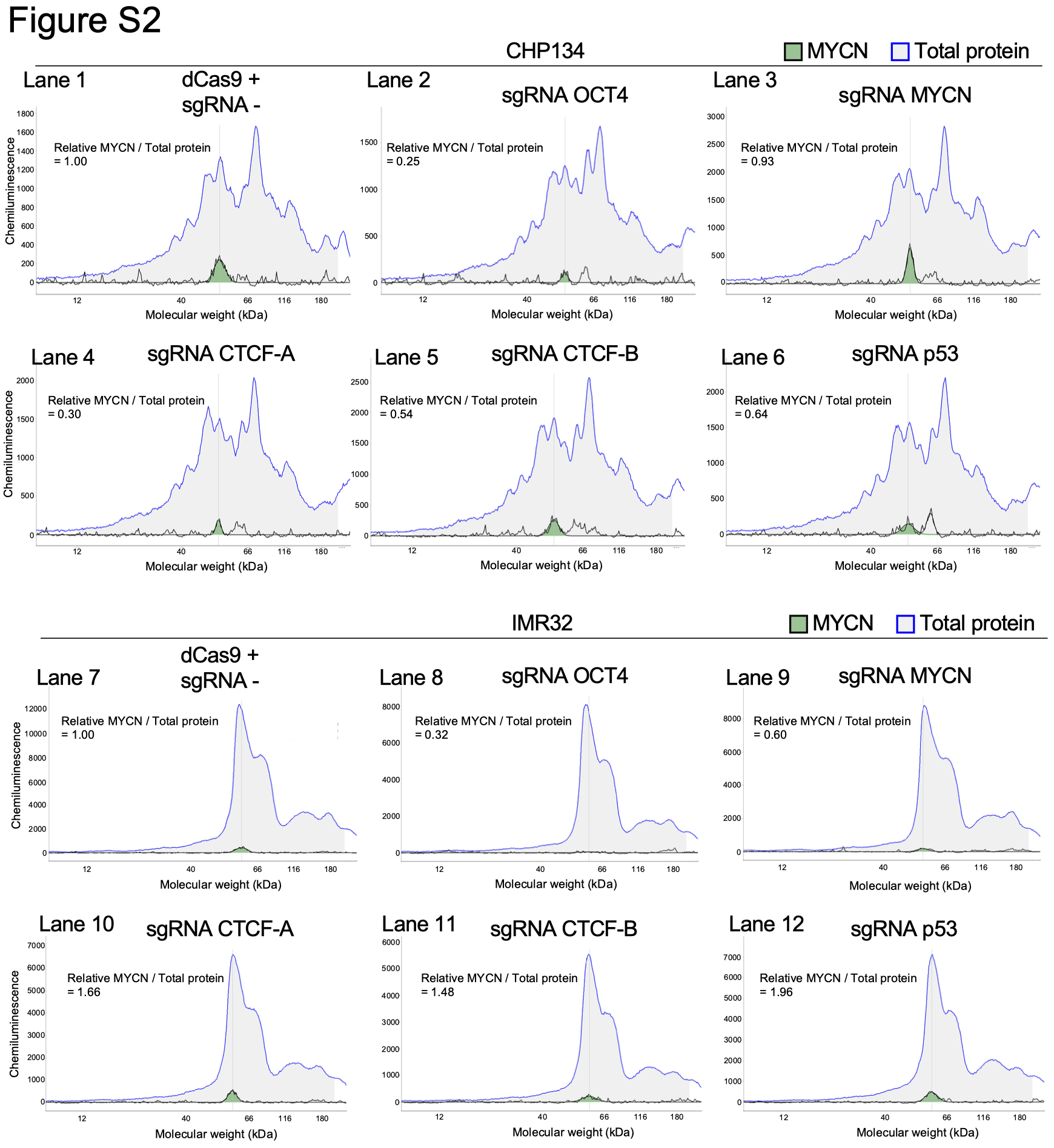

Supplement: Supplementary file 3 [file Image_2.tiff]

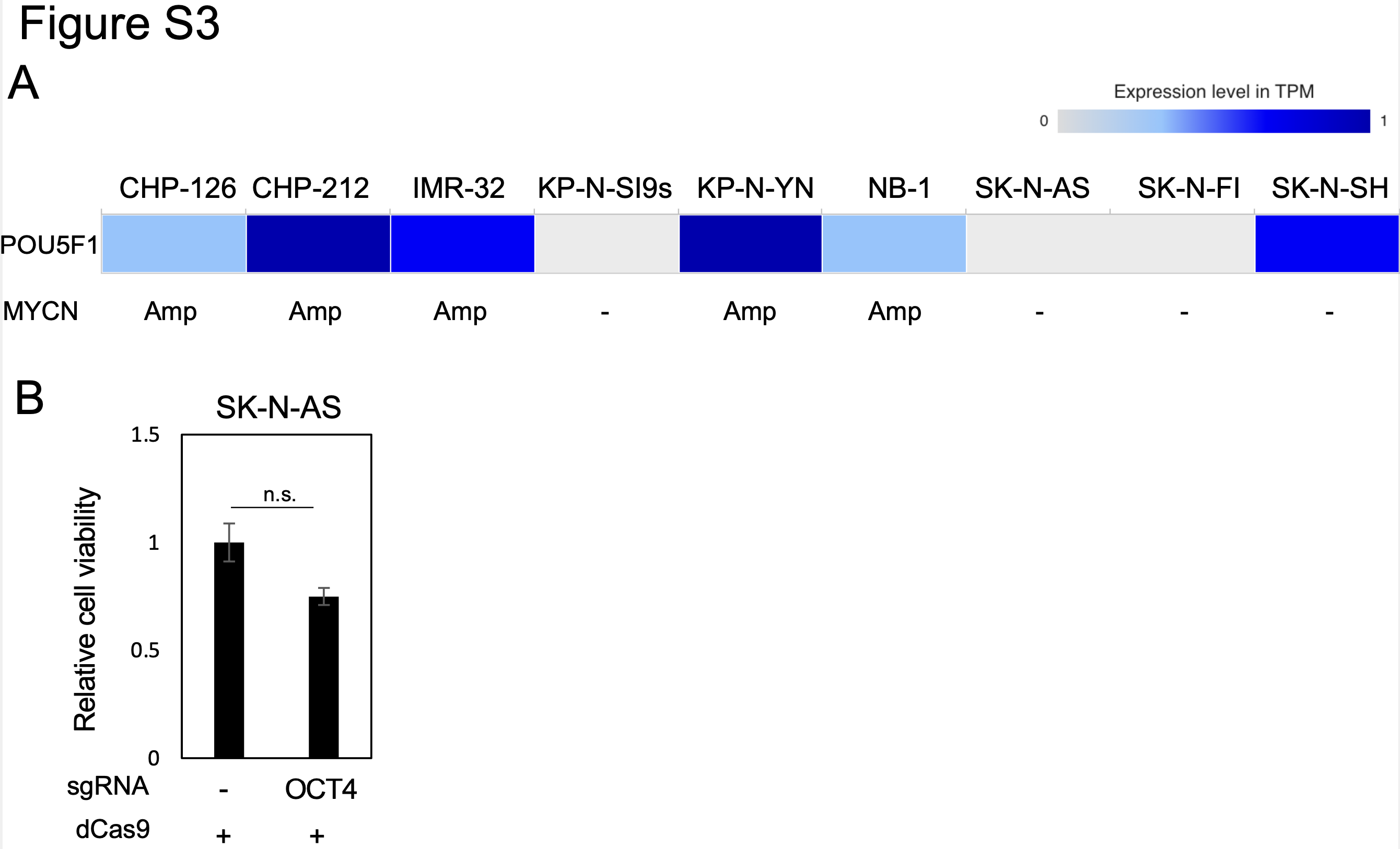

Supplement: Supplementary file 4 [file Image_3.tiff]

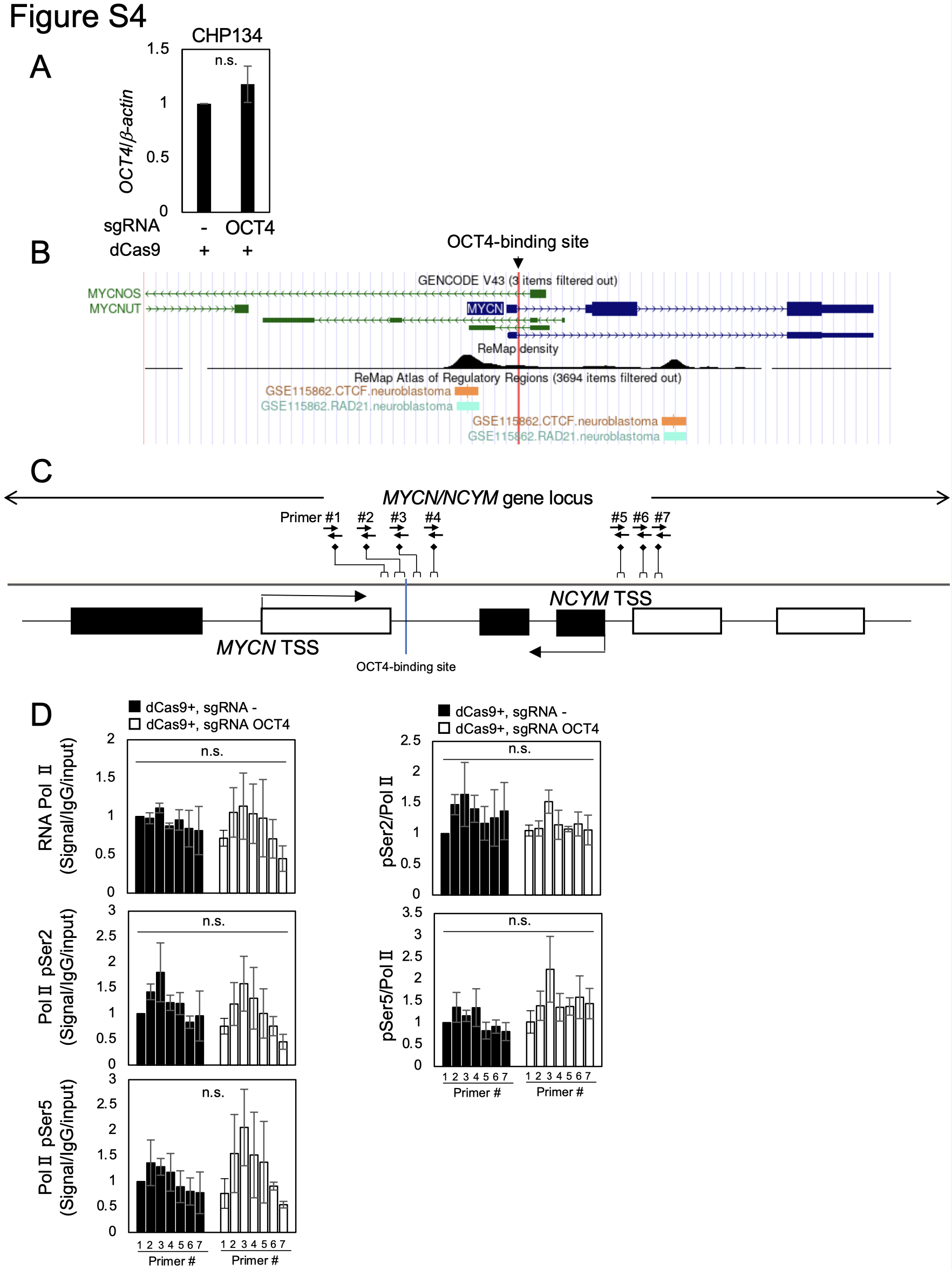

Supplement: Supplementary file 5 [file Image_4.tiff]

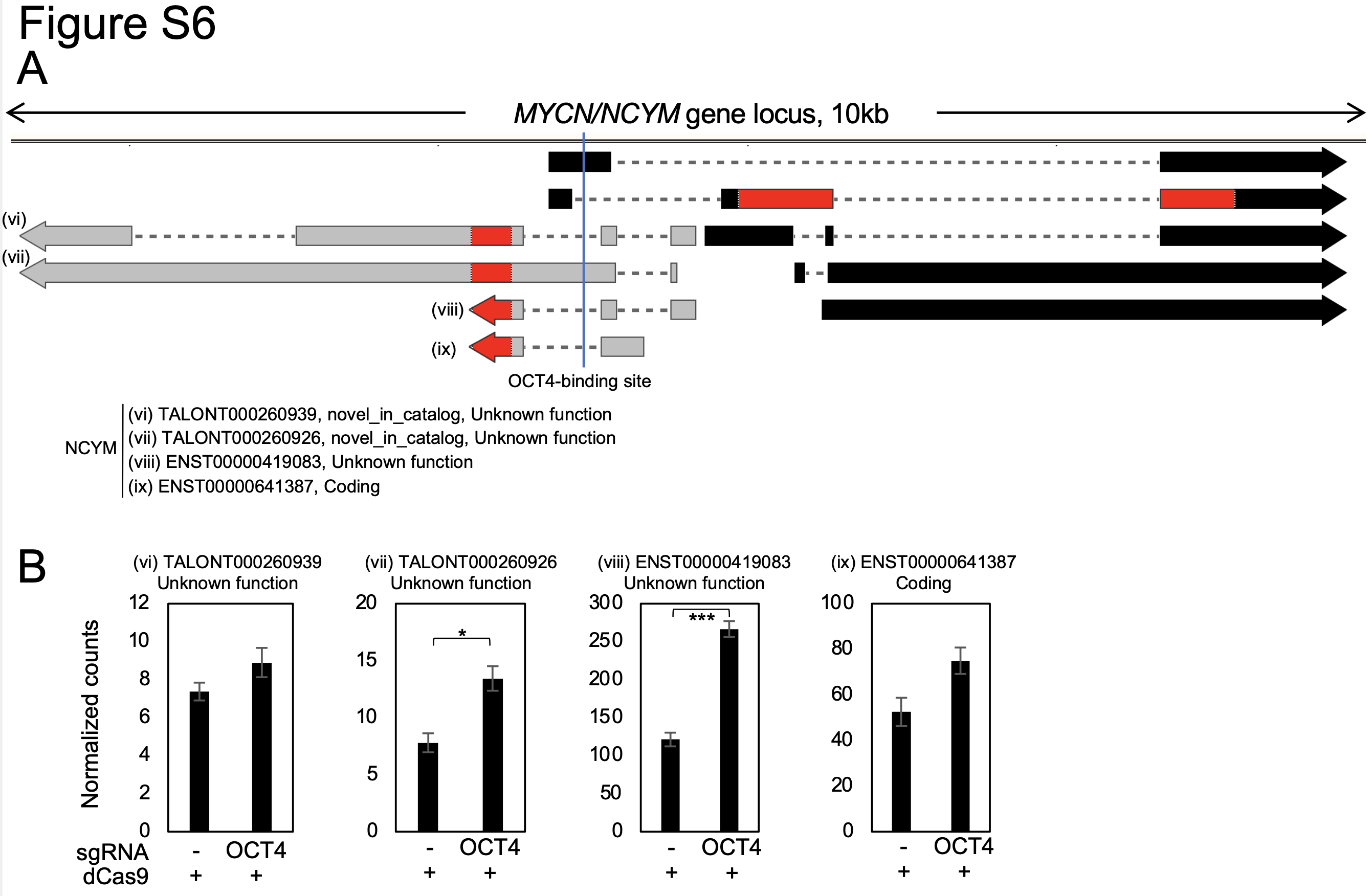

Supplement: Supplementary file 7 [file Image_6.tiff]

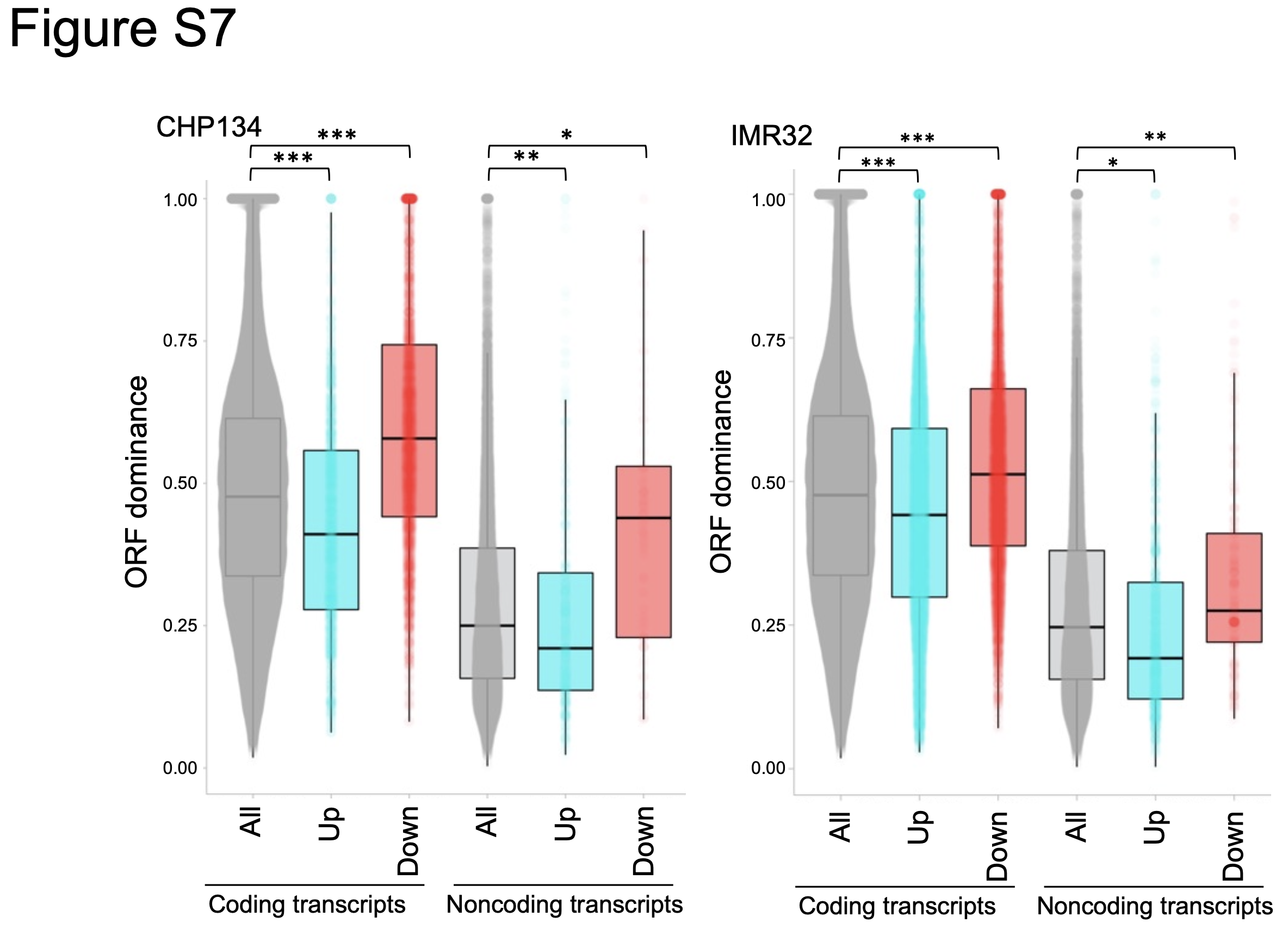

Supplement: Supplementary file 8 [file Image_7.tiff]
